# Uncharted territory: the arrival of *Psychoda albipennis* (Zetterstedt, 1850) (Diptera: Psychodidae) in Maritime Antarctica

**DOI:** 10.3389/finsc.2024.1481444

**Published:** 2024-12-17

**Authors:** Jordan Hernandez-Martelo, Tamara Contador, Sanghee Kim, Carla Salina, Claudia S. Maturana, Manuel Suazo, Peter Convey, Hugo A. Benítez

**Affiliations:** ^1^ Millennium Institute Biodiversity of Antarctic and Sub-Antarctic Ecosystems (BASE), Santiago, Chile; ^2^ Cape Horn International Center (CHIC), Centro Universitario Cabo de Hornos, Puerto William, Chile; ^3^ Laboratorio de Ecología y Morfometría Evolutiva, Centro de Investigación de Estudios Avanzados del Maule, Universidad Católica del Maule, Talca, Chile; ^4^ Programa de Doctorado en Salud Ecosistémica, Centro de Investigación de Estudios Avanzados del Maule, Universidad Católica del Maule, Talca, Chile; ^5^ Núcleo Milenio de Salmónidos Invasores (INVASAL), Concepción, Chile; ^6^ División of Life Sciences, Korea Polar Research Institute, Incheon, Republic of Korea; ^7^ Departamento Científico, Instituto Antártico Chileno, Punta Arenas, Chile; ^8^ Instituto de Alta Investigación, Universidad de Tarapacá, Arica, Chile; ^9^ British Antarctic Survey (BAS), Natural Environment Research Council, Cambridge, United Kingdom; ^10^ Department of Zoology, University of Johannesburg, Auckland Park, South Africa; ^11^ School of Biosciences, University of Birmingham, Edgbaston, Birmingham, United Kingdom

**Keywords:** biological invasion, non-native species, insects, flies, Antarctica, moth flies

## Abstract

Despite increasing awareness of the threats they pose, exotic species continue to arrive in Antarctica with anthropogenic assistance, some of which inevitably have the potential to become aggressively invasive. Here, we provide the first report of the globally cosmopolitan species *Psychoda albipennis* (Diptera, Psychodidae; commonly known as moth flies) in Antarctica during the austral summer of 2021/2022, with the identification confirmed using traditional taxonomic and molecular approaches. The species was present in very large numbers and, although predominantly associated with the drainage and wastewater systems of Antarctic national operator stations in synanthropic situations, it was also present in surrounding natural habitats. While it is unclear if *P. albipennis* is capable of long-distance dispersal, adult psychodid flies are known to travel more than 90 m from their emergence sites, and up to 1.5 km with wind assistance. Thus, once established in the natural environment of King George Island there appears to be a high risk of the species rapidly becoming invasive. The introduction of non-native species such as *P. albipennis* can be a significant driver of future biodiversity change and loss, and seriously impact ecosystem health. In vulnerable low diversity ecosystems, such as in the terrestrial environments of Antarctica, non-native species can lead to step changes in ecological functions and interactions, displace native species and, potentially, lead to the extinction of native biota.

## 1 Introduction

Throughout their evolution, humans have migrated to and colonized most regions of the world, with this movement drastically accelerating during recent centuries and decades through the development of new technologies and the globalization of trade and tourism activities (1). However, it was not until the last one to two centuries that humans started to have contact with Antarctica, and the continent remains the only one that has never had a native human population. Today, the Antarctic continent’s ecosystems remain amongst the least disturbed on Earth, with a long history of evolutionary isolation, and are characterized by low biological diversity, high levels of endemism and communities with low interspecific competition (2, 3). Its extreme climatic conditions and considerable geographic isolation have largely protected it against natural colonization processes (4–6), with no natural colonists known to have successfully established in Antarctica in the last one to two centuries of human contact with the continent and only two putative natural colonists in the sub-Antarctic since first human contacts in the Eighteenth Century (7).

Despite this level of natural protection, the combined effects of contemporary anthropogenic climate change and the growth of human activity on and around the continent are now facilitating the introduction of non-native species to Antarctica, highlighting the vulnerability of its ecosystems (7–13). Non-native species are recognized as one of the primary drivers of biodiversity loss globally, with their presence typically linked to the degradation of ecosystem health (14), and have been recognized as a particular threat to Antarctic terrestrial ecosystems (4, 8). They can lead to the introduction of new ecological functions and interactions, the loss of ecosystem services, the displacement of native species, and, ultimately, the extinction of native biota (15–19). Ecosystems characterized by low diversity and simple community structure, as is the case in Antarctic terrestrial ecosystems, are considered to be particularly vulnerable (10, 20).

Antarctic terrestrial biodiversity is characterized by a high degree of endemism, with terrestrial fauna consisting mainly of microarthropods (predominantly mites and springtails) and microinvertebrates (nematodes, tardigrades and rotifers) (3, 21, 22). Compared to other regions of the world, this biodiversity remains relatively unaffected by human activities. However, since the initiation of human contact with the sub-Antarctic in the Eighteenth Century, the Antarctic Peninsula in the early Nineteenth Century and the main body of the continent at the end of the Eighteenth and start of the Nineteenth Centuries, anthropogenic activities have contributed to a rapid increase in the arrival and establishment of non-native species across the region, with currently over 200 species established on the sub-Antarctic islands, at least 15 in the Antarctic Peninsula/maritime Antarctic, and records of many more arriving but not subsequently establishing, of which approximately 30% are insects (4, 10, 23, 24). The threats arising from non-native species establishment have raised considerable concern in the Antarctic Treaty Consultative Meetings (the governing mechanism of Antarctica under the Antarctic Treaty System) (8), as well as interest in the context of biological invasions. The Antarctic continent hosts only two native species of holometabolous insects, the winged Antarctic chironomid midge (*Parochlus steinenii* (Gercke, 1889)) and the wingless midge (*Belgica antarctica Jacobs, 1900*), the latter being paleoendemic to the Antarctic Peninsula and South Shetland Islands (23, 25).

In maritime Antarctica, which includes the Antarctic Peninsula (APR) and the Scotia Arc archipelagos of the South Shetland Islands, South Orkney Islands and South Sandwich Islands, one of the first reports of an established non-native invertebrate species was that of the midge *Eretmoptera murphyi* (Schaeffer, 1914) (Chironomidae, Orthocladiinae), native and endemic to sub-Antarctic South Georgia, on Signy Island (South Orkney Islands) (23, 26). It is thought to have been introduced in the 1960s as a result of plant transplantation experiments, although it was first observed and formally reported in the 1980s (27, 28). Its larvae are detritivorous, breaking down dead moss material, and recently being described as ecosystem engineers on Signy Island as they appear to be responsible for a nearly order of magnitude increase in the breakdown of moss peat and up to a five-fold increase in the release of available nitrogen (29). The dipteran *Trichocera maculipennis* Meigen, 1818 (Trichoceridae), a species whose larvae are detritivorous and also coprophagous, has been established with an increasing distribution on King George Island, South Shetland Islands, since the austral summer of 2006/2007 (30, 55, 56). Various species have been reported in synanthropic situations, generally associated with national research stations, either on single occasions or, in a small number of instances, becoming established on those stations for multiple years even in the face of eradication efforts, for instance, the fly of Genus *Lycoriella* on Australia’s Casey Station on the continental Antarctic coast (31). Most recently, the synanthropic presence of the food storage pest *Plodia interpunctella* (Indian meal moth) has been reported at Comandante Ferraz and Yelcho Stations in the South Shetland Islands and north-west Antarctic Peninsula (57, 58).

**Figure 1:**
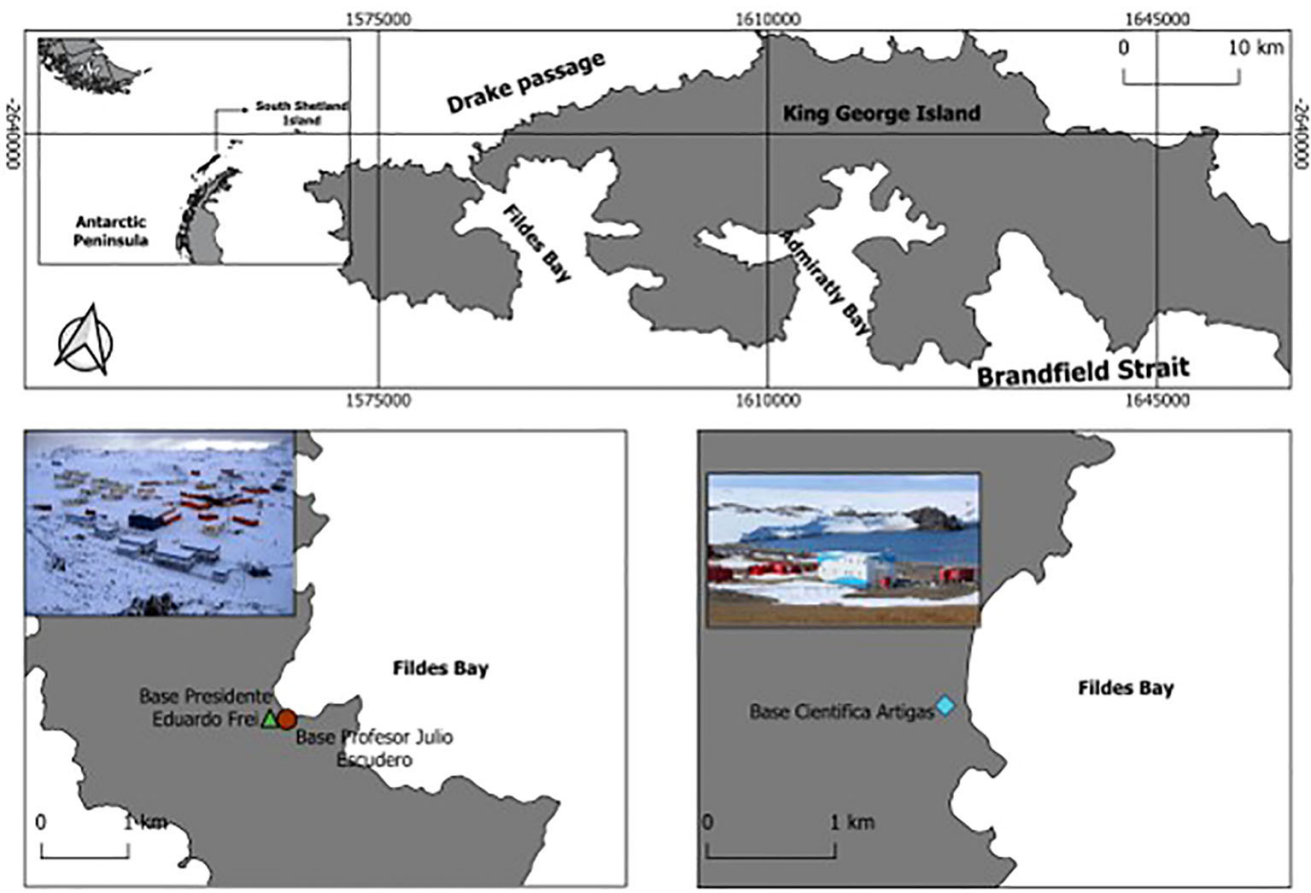
Map of King George Island indicating the location of the two scientific stations where *P. albipennis* was first observed and collected in the 2021/22 austral summer season.

The aim of this study is to provide the first formal report of the presence of the non-native moth fly, Psychoda albipennis Zetterstedt, 1850 (Diptera, Psychodidae), in Antarctica, documented through repeated sightings during the austral summer seasons of 2019/20, 2021/22, 2022/23, and 2023/24 (**Figure 1**).

## 2 Materials and methods

### 2.1 Sample collection

**Figure 2:**
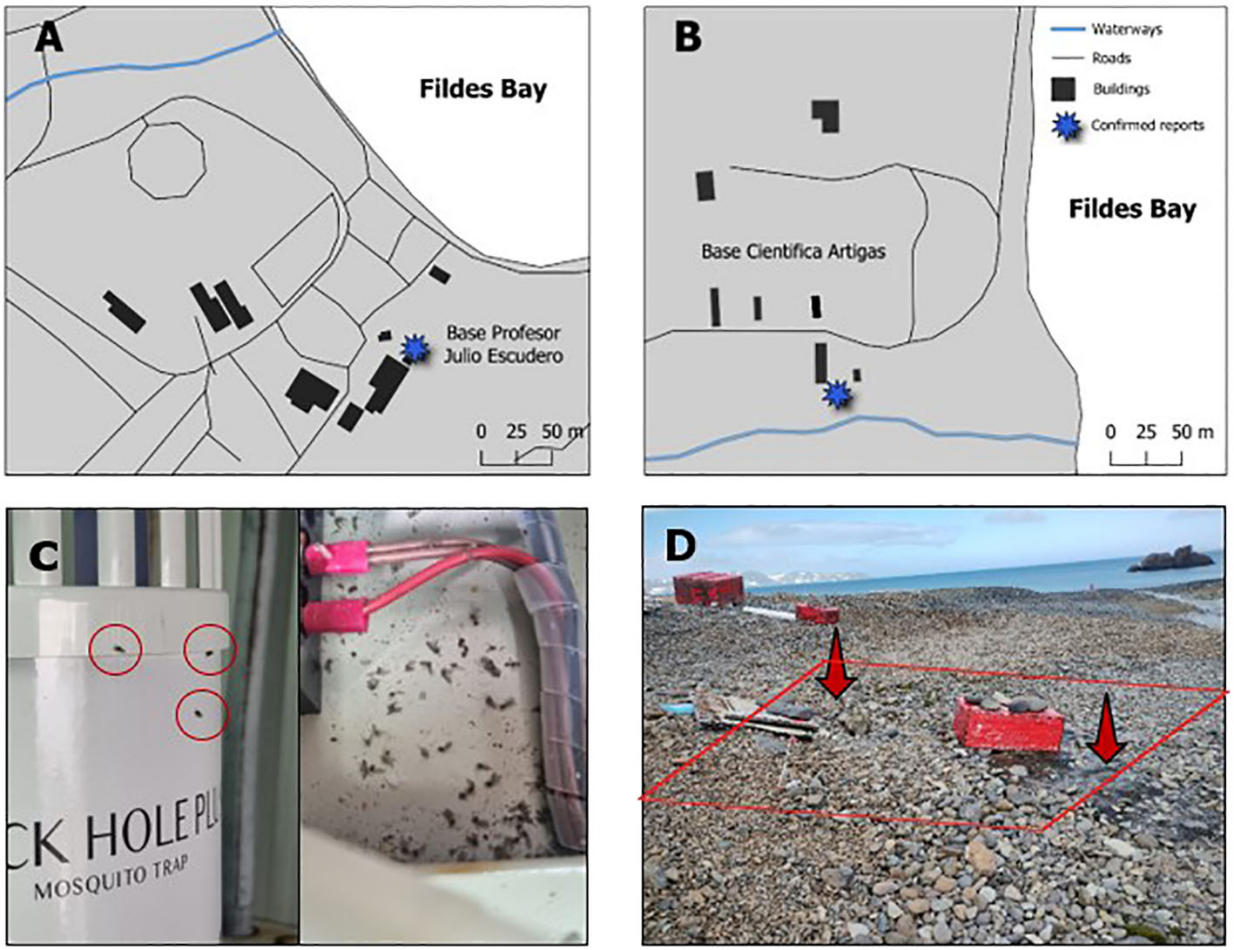
Antarctic stations where synanthropic establishment of *P. albipennis* has been reported. **(A)** Drainage systems of Base Julio Escudero. **(B)** Drainage systems of Base Scientific Artigas. **(C)** Individuals of *P. albipennis* in the treatment plant of Base Escudero. **(D)** The external drainage system of the Artigas Scientific Base, leaks were observed, and the presence of flies was detected in the immediately surrounding area.

During the austral summer of 2021/2022, the presence of *Psychoda albipennis* Zetterstedt, 1850 was noted for the first time in the sewage treatment system of the Chilean Julio Escudero Station, on King George Island (32). During this season, as well as in the subsequent 2022/23 and 2023/24 seasons, *in situ* observations and monitoring of the status of the species *P. albipennis* were conducted. Additionally, 17 and 15 flies were collected from sewage treatment facilities at the Uruguayan Artigas and Chilean Escudero stations, respectively (**Figure 2**), using UV traps and entomological aspirators, and stored in 90% ethanol. These 32 flies were used to obtain total DNA, in order to confirm species identity.

### 2.2 Fly identification

Initial taxonomic identification was carried out with reference to the taxonomic keys Withers (33) and Coe et al. (34). This was then confirmed using molecular analyses.

#### 2.2.1 DNA extraction

Each of the 32 flies was individually homogenized in 200μL of Solid Tissue Buffer (TissueLyser II, Qiagen, Germany) supplied in the Quick-DNA Microprep Plus Kit (Zymo Research, USA). The specimens were then digested at 55°C for 1 h. Total genomic DNA was then extracted using the Quick-DNA Microprep Plus Kit (Zymo Research, USA) following the manufacturer’s instructions. The resulting filter membranes were cut using sterile scissors and vortexed with 400μL of Solid Tissue Buffer for 1 min.

#### 2.2.2 PCR amplification and sequencing of mitochondrial molecular barcode

The cytochrome c oxidase subunit I (cox1) of mitochondrial DNA was amplified using standard primer pairs (35) using Phusion High-Fidelity PCR master mix (NEB, USA) in a Mastercycler (Eppendorf, Germany) under the following conditions: initial incubation at 98°C for 30 sec, followed by 38 cycles of denaturation at 98°C for 10 sec, annealing at 55°for 30 sec and extension at 72°C for 40 sec and, finally, 72°C for 10 min. The PCR products were purified using the QI quick PCR purification kit (Qiagen, Germany) and sequenced using BigDye 1.1 terminator cycle sequencing reagents on an ABI PRISM 3130 Automated Capillary DNA sequencer (Applied Biosystems, USA).

#### 2.2.3 Phylogenetic analysis and BLAST

In order to assess similarity between our newly generated and publicly available sequences of *Psychoda albipennis*, we used the Basic Local Alignment Search Tool (BLAST) from the GenBank server (http://www.ncbi.nlm.nih.gov/Genbank). The cox1 sequence alignments were generated using Geneious software (36). The phylogenetic tree was generated by a Maximum Likelihood (ML) method under GTR model carried out using RAxML v8.2.12 (37). The degree of DNA divergence was calculated in DNAsp v6 (38) between the different clades/cluster that we may find after the phylogenetic reconstruction.

Accession numbers for each novel nucleotide sequence of cox1 generated in this study are given in **Supplementary Table 1** (see also **Supplementary Material**)

## 3 Results

### 3.1 Field observation

*Psychoda albipennis* was first observed during the austral summer of 2019/2020 at the treatment plant facilities of the Chilean Frei Base as a result of monitoring directed at *T. maculipennis* (**Figure 2A**). The taxonomic identification of the fly was carried out in 2021 at the Korea Polar Research Institute using DNA barcoding. In the 2021/22 season, *P. albipennis* flies were reported in the treatment system of King Sejong Station (32).

During the 2022/23 and 2023/24 monitoring periods, a large number of *Psychoda albipennis* individuals were observed alongside *Trichocera maculipennis* around the drainage system of the Artigas Scientific Base (**Figures 2B, D**; see **Supplementary Video 1**). Due to the strong winds, the *P. albipennis* individuals were found under rocks or among mosses near the drainage system. Occasionally, the wind carried them several meters away from the drainage system. They were generally quite active and were often seen walking beneath the rocks. At the Escudero Base, the presence of individuals in the drainage system was reported by the base’s logistical staff. Specimens were observed both in UV traps and in various areas within the wastewater treatment room. It was noted that ambient temperature significantly influenced the activity of the flies: as the temperature increased, their mobility increased markedly, whereas a decrease in temperature resulted in a reduction of their motor activity (**Figure 2C**). To date, *P. albipennis* has not been directly observed in natural environments outside the vicinity of Antarctic stations during monitoring, possibly due to the lack of adequate bioprospection. Despite this, by the end of the 2022/23 season, the presence of “bath flies” was detected in moss samples collected from Arley Island, King George Island (Gustavo Zúñiga, pers. comm.) (see **Supplementary Video 2**).

### 3.2 Taxonomic identification

**Figure 3:**
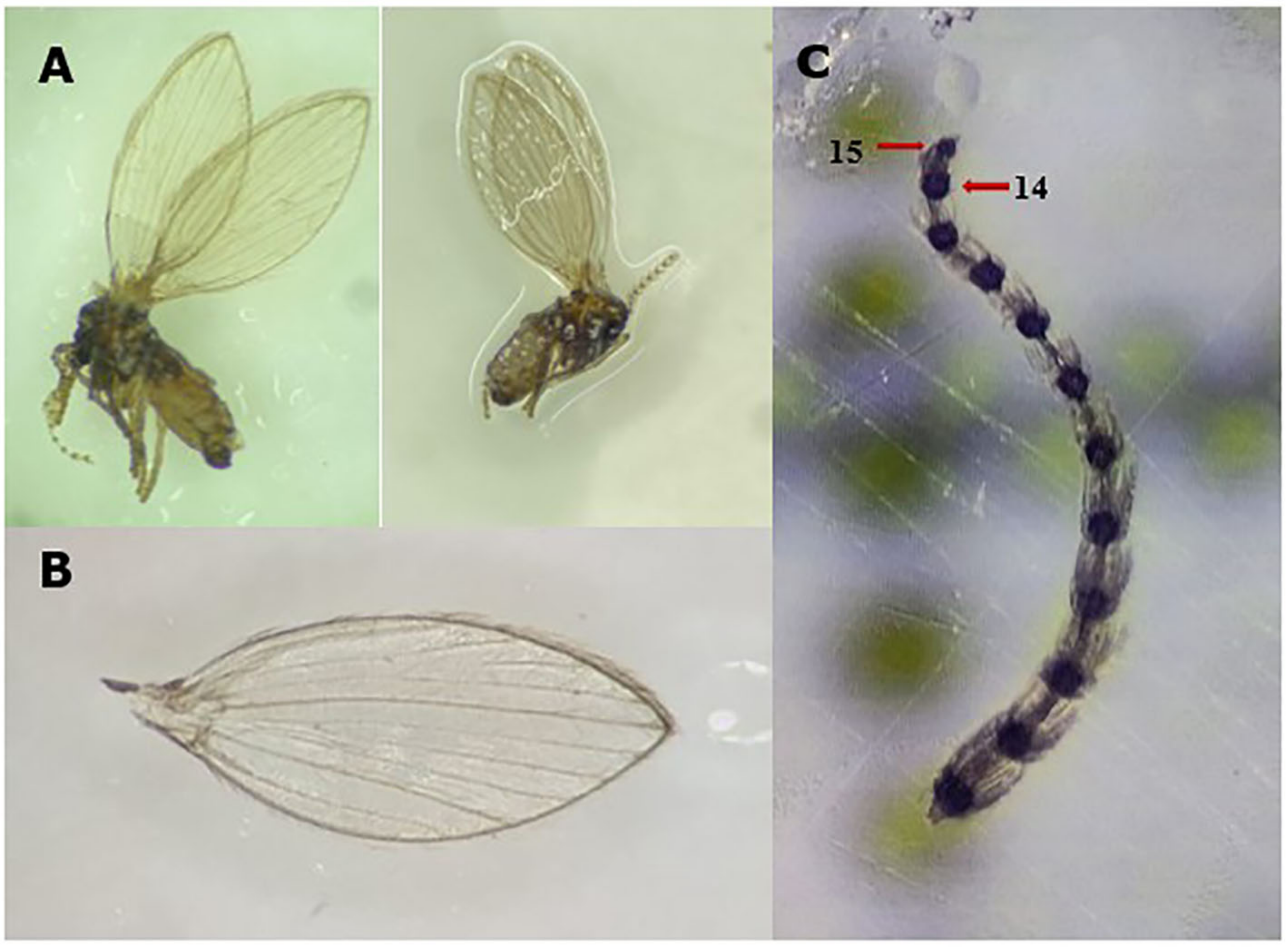
Key morphological features of *P. albipennis*: **(A)** Adult *P. albipennis*, showing the pale white color. **(B)** Wing of *P. albipennis*, showing the absence of black spots at the ends of the veins. **(C)** Antenna of *P. albipennis*, showing that the fourteenth segment is larger than the fifteenth. Photography by Rocío Oróstica.

The distinctive morphological features of adult *P. albipennis* were first described by Coe et al. (34). The absence of dark spots at the ends of the veins, the shape of the antennal tips, and the pale whitish gray color (**Figures 3A–C**) were used to confirm the identity of the specimens collected on King George Island.

### 3.3 Molecular identification and phylogenetic analysis

**Figure 4:**
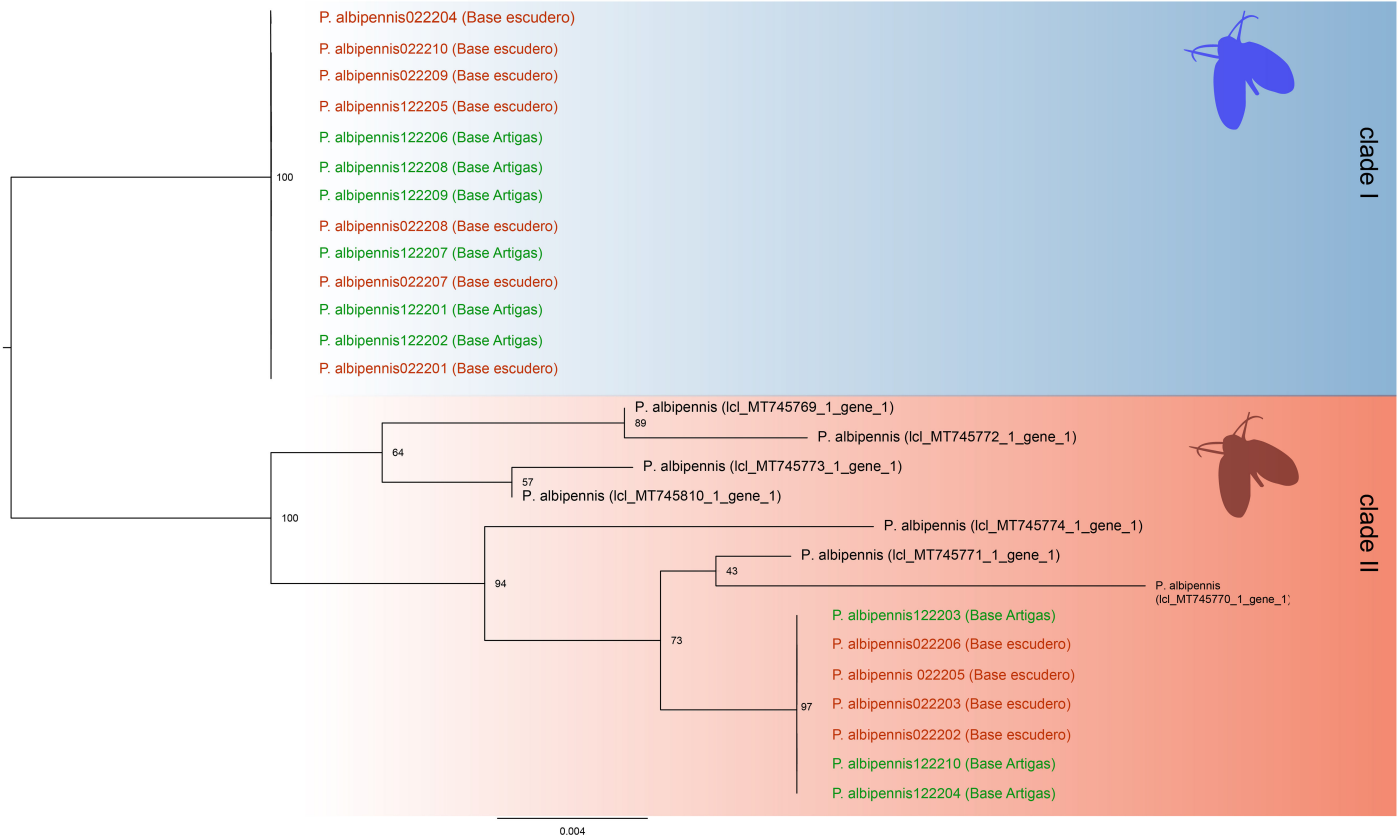
Maximum Likelihood phylogenetic relationships between Antarctic and GenBank-available European samples of *P. albipennis* Clade I comprises only new samples obtained from King George Island. Clade II includes all the sequences obtained from GenBank as well as the remaining new sequences from King George Island.

We obtained cox1 sequences of 650 bp of from 20 of the 32 specimens of moth flies collected on King George Island. The resulting cox1 consensus sequences, after BLAST alignment against the GenBank database, provided a 98% match to the *P. albipennis* mitochondrial cox1 sequence (accession number: MT745810.1, **Figure 4**).

In the molecular phylogenetic analysis using Maximum Likelihood we included seven available sequences representing *P. albipennis* (**Figure 4**). We detected two clades with 2.3% of divergence (15 nucleotide differences between clades), and strong bootstrap support. Clade I included sequences from both Antarctica Research stations specimens, while Clade II included our sequences from both Antarctica Research stations too and GenBank available sequences from Europe two clades).

## 4 Discussion

This study presents the first report of a member of the family Psychodidae in Antarctica, *Psychoda albipennis*, with identification confirmed through both morphological and molecular methods. Some specimens of this species from Artigas and Escudero research stations in Antarctica were present in one of the two clades identified along with multiple sequences of European origin. The two clades containing Antarctic *P. albipennis* were clearly divergent, but more loci and sampling locations are required to confirm whether this is an indication of different source of species introduction.

Members of the genus *Psychoda* are commonly known as moth flies. They were observed for the first time in synanthropic locations around buildings of stations operated by two Antarctic national operators during the austral summer of 2021/22, with continued presence confirmed during the subsequent austral summers of 2022/23 and 2023/24 (**Figure 1**). *Psychoda albipennis* is considered a globally cosmopolitan species, with the exception of Antarctica. It is a detritivorous species with saprophagous larvae and is often found in synanthropic locations and their environs (39, 40). It is often associated with humid or semi-humid environments (puddles, ditches, tree hollows) and in highly organic enriched environments such as drainage and wastewater treatment systems, consistent with the observations here in sewage treatment systems of Antarctic national operator stations (**Figure 2C, D**) (40, 41).

*Psychoda albipennis* is a holometabolous species, with a life cycle involving four stages (egg, larva, pupa, adult) the duration of which depends on environmental conditions (42, 43). While detailed studies of the life cycle of this species are not available, those of some congeneric species allow some inference to be made about factors underlying its successful establishment in Antarctica. Griffith and Gillett-Kaufman (44) indicate that females of *P. alternata* (Say, 1824) lay their eggs on moist soil or near pipes and sewage systems, as well as directly on fecal matter and decomposing plant material (41, 45). The larval stage is the longest, including four larval instars each with a duration of 9 to 15 d at 21°C (42). Both the eggs and larvae are capable of entering diapause, which could potentially enable *P. albipennis* to hibernate and survive the Antarctic winter. However, confirming this would necessitate physiological studies (40, 44). Moth flies are considered poor fliers. However, they are capable of short flights and can move up to 90 m from their emergence site, and can also be displaced up to 1.5 km by wind (40). In the context of their occurrence on/around research stations on King George Island, this highlights the risk of dispersal and colonization of natural Antarctic habitats, such as seal breeding, molting and resting areas, where the high density of organic matter and fecal matter present would provide an ideal food source.

*Psychoda albipennis* is not considered an exotic species elsewhere globally and there are no reports to date of its impacts on natural systems. However, understanding its biological characteristics can shed light on the risks this species may pose in Antarctica through introducing new ecological interactions and functions into such fragile ecosystems. Bartlett et al. (29) reported that the presence of the non-native chironomid, *Eretmoptera murphyi*, on maritime Antarctic Signy Island (South Orkney Islands) can strongly influence decomposition rates, with potential knock-on impacts on native plant and invertebrate communities. It can also cause a 4 to 5-fold increase in the availability of inorganic nitrogen in the habitats it occupies, highlighting the problems that can result from the introduction of new ecological interactions into Antarctic ecosystems.

The terrestrial arthropod fauna of the Antarctic continent consists predominantly of micro-arthropods with only two species of true Diptera, the chironomid midges *Parochlus steinenii* and *Belgica antarctica* (21, 26). The presence of non-native species, especially those that can overcome ecological filters (biotic and abiotic factors) and achieve biological functions (such as reproduction) (46), should be considered a threat to Antarctic ecosystems and their biological diversity (10, 47). Due to this threat, the development and implementation of policies to minimize the risk of further species being imported to Antarctica control those that are already established have been promoted. These include the adoption of strict regulations under the Protocol on Environmental Protection to the Antarctic Treaty (known as the Environmental Protocol and provision of detailed practical advisory guidelines through the COMNAP Non-Native Species Manual, such as the prohibition of the intentional introduction of non-native species (with exceptions subject to rigorous permits) and the promotion of scientific spaces with experts to develop strategies that help minimize the risks of accidental introduction to the continent (47–49).

The most effective (and cost-effective) goal of any biosecurity strategy should be to prevent the arrival of a non-native species in the first place (48, 50), To provide a solid foundation for any effective biosecurity system, timely information on newly arriving non-native species and those already present must be available (51). It is axiomatic that early detection is more likely to allow rapid and effective action to control the spread before an invasion becomes too extensive to be practically controlled (51, 52). However, despite the apparent widespread recognition of the threats to Antarctic ecosystems and species posed by the establishment of non-native species, several examples, even after the Environmental Protocol came into force, highlight that Antarctica remains far from being effectively protected today.

Although prevention and timely reporting are crucial for a rapid and effective response to the accidental introduction of exotic species, it is equally important for scientific and military bases in the area governed by the Antarctic Treaty to continually improve their wastewater treatment systems and disposal methods. This is essential to prevent leaks and discharges of inadequately treated organic or inorganic matter into the Antarctic environment. Such discharges can have implications for the health of Antarctic fauna, both marine and terrestrial (53). While it has not been proven that species introductions occur through this route, there is evidence suggesting that discharges contribute to the establishment and persistence of non-native species (30, 54).

In the current study, we present observations confirming the presence of *P. albipennis* in the Antarctic environment, both within the stations and in their immediate surroundings (**Figure 2C**). Additionally, *ex situ* observations of *P. albipennis* survival in mosses in natural environments not associated with scientific bases indicate that the species may have established and reproduced. This situation is of serious concern, as it may indicate that the species has a significant capacity for adaptation (physiological, genetic, morphological) and potential for establishment and invasion in other areas with environmental conditions similar to those of the Fildes Peninsula.

## Data Availability

The original contributions presented in the study are included in the article/**Supplementary Material**. Further inquiries can be directed to the corresponding author/s.
