# Supplementary material for: Uncharted territory: the arrival of Psychoda albipennis (Zetterstedt, 1850) (Diptera: Psychodidae) in Maritime Antarctica
Source: Front Insect Sci. 2024 Dec 17;4:1481444. doi: 10.3389/finsc.2024.1481444 (PMC11685106; doi:10.3389/finsc.2024.1481444)
Supplement: Supplementary file 1 [file Table1.docx]

| Marker Used | Accession number GenBANK |
| --- | --- |
| Cox1 | **MT745769** |
| Cox1 | **MT745770** |
| Cox1 | **MT745771** |
| Cox1 | **MT745772** |
| Cox1 | **MT745773** |
| Cox1 | **MT745774** |
| Cox1 | **MT745810** |
